# Bridging the nutrition guidance gap for GLP-1 receptor agonist therapy assisted weight loss: lessons from bariatric surgery

**DOI:** 10.1038/s41366-025-01952-w

**Published:** 2025-11-15

**Authors:** Marie Spreckley, Cara F. Ruggiero, Adrian Brown

**Affiliations:** 1 MRC Epidemiology Unit, University of Cambridge, Cambridge, UK; 2 Centre for Obesity Research, University College London, London, UK; 3 Bariatric Centre for Weight Management and Metabolic Surgery, University College London Hospital NHS Trust, London, UK; 4 National Institute for Health Research, UCLH Biomedical Research Centre, London, UK

**Keywords:** Nutrition, Weight management

## Introduction

Glucagon-like peptide-1 receptor agonists and combination incretin medications (hereafter referred to as GLP-1 RAs) represent a paradigm shift in the management of obesity, approaching weight loss outcomes comparable to bariatric surgery [1, 2]. Despite global utilisation, there remain critical clinical and research gaps in structured nutritional guidance for their use. These gaps present a clinical concern, since GLP-1 RA-induced weight loss mirrors several physiological effects of bariatric interventions, potentially resulting in significant reductions in lean mass, micronutrient depletion, altered eating behaviours, gastrointestinal symptoms, and gallstone formation [3].

While physical activity and behavioural interventions are integral to long-term weight management, this discussion focuses on the currently neglected domain of nutrition in the context of GLP-1 RAs. We present a novel perspective by integrating evidence on nutritional risks associated with GLP-1 RAs with established post-bariatric dietary frameworks, to identify actionable insights and areas of uncertainty, ultimately arguing for the urgent development of international GLP-1 RA-specific nutrition guidelines in adult populations (Table 1). References were selected through expert consensus based on relevance to obesity pharmacotherapy and post-bariatric models of care.

**Table 1:** Nutritional Considerations with GLP-1 RA Therapy vs Bariatric Surgery.

Nutritional Parameter	Bariatric Surgery Mechanisms	GLP-1 RA Mechanisms	Bariatric-Based Nutritional Strategies
Lean Mass Loss and Sarcopenia	Malabsorption and energy restriction accelerate lean mass loss	Energy deficit and reduced protein intake contribute to lean mass loss	Higher protein intake from high-quality sources; early prioritisation of protein; monitoring for sarcopenia, especially in older adults and those at risk of or with sarcopenic obesity. Between 0.8–1.6 g/kg/d or absolute protein amounts (80–120 g/day)
Micronutrient Deficiency	Reduced absorption of iron, B12, vitamin D, calcium, thiamine	Reduced intake and possible dietary quality due to early satiety, nausea, and food restrictions	Pre-treatment micronutrient screening; routine lifelong complete multivitamin and mineral supplementation (including iron, B12, calcium, zinc, copper and folic acid); proactive monitoring for deficiencies every 6 months
Altered Food Preferences	Changes in taste, aversions to meats and fats	Decreased interest in nutrient-dense foods, especially protein- and fat-rich foods	Behavioural nutrition therapy to address food-related cognition, sensory experience, and emotional eating; patient education on food choices and preferences; referral to psychological support, if required.
Gastrointestinal Symptoms	Nausea, vomiting, early satiety, diarrhoea, dumping syndrome	Nausea, vomiting, constipation, particularly during dose titration and higher doses	Small, frequent meals; slow eating; fluid separation from meals; bland, low-fat foods during nausea; consider food containing ginger; increase fibre and fluid intake; consider use of medication if required.
Gallstone Formation	Rapid weight loss increases risk of gallstones due to gallbladder stasis and cholesterol supersaturation	Similar rapid weight loss trajectories; gallstones may form due to similar mechanisms	Moderate fat intake; ensure adequate hydration and fibre intake; consider ursodeoxycholic acid prophylaxis in high-risk cases, particularly for rapid treatment responders
Nutritional Monitoring	Regular biochemical surveillance and dietitian-led follow- up; routine screening for deficiencies and adjustments	No routine nutritional monitoring in clinical practice, leading to potential undiagnosed deficiencies	Implement structured nutritional follow-up with regular biochemical testing (e.g., B12, iron, calcium); integrate dietetic support into clinical pathways, especially for long-term therapy

A comparative overview of physiological mechanisms, nutritional risks, and management strategies in GLP-1 RA Therapy and bariatric surgery, based on BOMSS guidance and clinical literature [1, 6–8, 10].

## GLP-1 RA therapy induced weight loss: nutritional risks and lessons from bariatric surgery

### Preservation of lean mass and protein intake

GLP-1 RAs suppress appetite and reduce food cravings, with trials showing up to 22.5% mean weight loss at 72 weeks [1]. Although this facilitates significant fat mass reduction, an estimated 30–40% of weight lost may derive from fat-free mass, which is of particular concern for older adults and individuals with sarcopenic obesity [4, 5]. While some evidence suggests GLP-1 RAs may spare lean mass more effectively than alternative strategies [1], this is not universally observed. Loss of skeletal muscle can impair metabolic health, diminish physical function, and compromise weight maintenance [6]. Drawing on bariatric protocols, high-quality protein intake should be prioritised for adults with obesity using GLP-1 RAs [7], ideally accompanied by resistance training. Presently, there is a lack of prospective studies to determine optimal protein amounts, though ranges between 0.8–1.6 g/kg/d or absolute protein amounts (80–120 g/day), similar to those proposed for bariatric surgery, have been proposed [8].

Therefore, urgent research gaps include ideal dosing during GLP-1 RA use, and the role of protein and resistance training in minimising loss of lean mass. In addition, appropriate measures need to be used to assess lean mass and functionality such as dual-energy X-ray absorptiometry, nitrogen balance, or handgrip strength. In studies focusing on the preservation of muscle mass, direct measurement with Magnetic Resonance Imaging should be prioritised.

### Micronutrient deficiencies and pre-treatment assessment

Early satiety, nausea, and changes in food preferences often result in reduced dietary variety and diminished intake of essential nutrients, including iron, vitamin B12, vitamin D, calcium, and thiamine [6, 9]. Although GLP-1 RAs do not induce malabsorption, the potential risk of micronutrient insufficiencies remains considerable due to low oral intake that may be nutritionally inadequate, as supported by recently published evidence [10]. Unlike bariatric surgery, where preoperative screening for micronutrient deficiencies is standard practice [6], no formal consensus recommendations currently exist for individuals commencing GLP-1 RAs. Baseline nutritional assessments, including dietary reviews and biochemical monitoring with a multidisciplinary team, are especially important for high-risk groups, particularly older adults and people who menstruate. A risk-based approach is preferable where feasible. However, routine supplementation with a once daily complete multivitamin and mineral supplement containing iron, zinc, copper, B12 and folic acid, may offer a pragmatic, interim strategy in the absence of standardised nutritional screening [7].

### Alterations in eating behaviour and behavioural support

Patients using GLP-1 RAs may experience changes in eating behaviour, including reduced hunger, fewer food cravings, and a decrease in binge eating, ‘food noise’, emotional eating, and uncontrolled eating [1]. These behavioural adaptations, while partially responsible for weight loss, could lead to suboptimal dietary quality. Nutrition interventions should address food-related cognition, sensory experience, and emotional eating.

Behavioural strategies may improve eating behaviours, dietary quality and adherence during GLP-1 RA use, although the longer-term psychological and behavioural impacts following medication cessation remain poorly understood and warrant further investigation [11]. Behavioural nutrition support is currently not standard practice when prescribing GLP-1 RAs and should be considered a core component of care to address unintended dietary and psychological consequences. For example, patients frequently report a reduction in intrusive food-related thoughts during GLP-1 RA use, referred to in some literature as ‘food noise’, recently defined and measured using a validated questionnaire [12]. Where required, behavioural nutrition support can be provided alongside more comprehensive psychological support during and after cessation of GLP-1 RA use.

Behaviour change frameworks such as the Capability, Opportunity, Motivation, Behaviour (COM-B) model can support the identification of psychological and contextual factors influencing behaviour [13]. Incorporating both COM-B principles and strategies to support food noise suppression into behavioural nutrition support may be a promising avenue to improve psychological wellbeing and dietary adherence.

### Gastrointestinal symptoms and structured meal practices

Nausea, vomiting, and constipation are commonly encountered side effects associated with GLP-1 RA use, particularly during dose titration and at higher doses [1]. Drawing on post-bariatric strategies, small, frequent meals, slow eating, and fluid separation may help mitigate symptoms [7]. Dietary adaptations such as favouring dry, low-fat foods during periods of nausea, increasing fibre and fluid intake may support treatment adherence. These pragmatic strategies, however, are currently inconsistently communicated to patients and remain absent from current GLP-1 RA care models.

### Gallstone risk and prophylactic measures

Rapid weight loss, irrespective of the mechanism, increases the risk of gallstone formation due to gallbladder stasis and cholesterol supersaturation. Gallstones occur in 10-30% of patients post-bariatric surgery [14], and similar trajectories in GLP-1 RA-induced weight loss suggest comparable susceptibility. Preventative strategies derived from surgical care, including moderating dietary fat, ensuring adequate hydration and fibre intake, and considering ursodeoxycholic acid in high-risk cases [7], should be explored in the context of GLP-1 RA treatment protocols.

### Monitoring and clinical follow-up

Structured nutritional follow-up, including regular dietary reviews and biochemical monitoring, is a hallmark of post-bariatric care [7]. In contrast, GLP-1 RAs are typically administered with minimal ongoing nutritional oversight. Without proactive monitoring, clinicians may fail to identify emerging deficiencies or suboptimal intake, particularly in patients receiving therapy outside specialist care settings. Establishing agreed monitoring protocols aligned with bariatric standards in consultation with nutrition professionals could enhance safety and treatment efficacy in this patient population.

## A call for GLP-1 RA therapy specific nutrition guidelines and prospective studies

The global, continuously growing adoption of GLP-1 RAs across public and private healthcare settings necessitates the development of comprehensive, international nutritional consensus guidance. Recent publications have begun to address nutritional considerations for obesity management medication treatment [8, 10], although there remains no global consensus framework for dietary screening, intervention, or supplementation for GLP-1 RA users. This void can lead to heterogeneity in clinical practice and may expose patients to preventable complications.

Nutrition therapy constitutes a foundational pillar of obesity treatment. Its exclusion from clinical GLP-1 RA care represents a critical oversight that may compromise patient outcomes. There is a need for the formation of an interdisciplinary task force, including experts in dietetics, psychology, obesity, endocrinology, surgery, and public health, to develop international GLP-1 RA-specific nutritional consensus guidelines. Guidelines should address screening protocols, macronutrient and micronutrient requirements, symptom management, behavioural support, and long-term follow-up to minimise risk and aid long-term weight maintenance. However, implementation challenges, including limited access to registered dietitians, lack of reimbursement for nutritional counselling, and broader structural inequities, must also be addressed to ensure equitable access to care.

## Conclusion

GLP-1 RAs are an important addition to the therapeutic toolkit for obesity management. However, their success is contingent not only on pharmacological efficacy but on comprehensive wraparound clinical support, particularly in nutrition. The physiological consequences of rapid weight loss, including lean mass loss, micronutrient deficiencies, altered eating behaviours, gastrointestinal symptoms, and gallstones, are well-documented. Clinicians should adopt a proactive approach by encouraging adequate protein intake and dietary quality, providing practical behavioural nutrition support, considering appropriate supplementation, and referring patients to registered dietitians. Nutrition must no longer remain a missing pillar in obesity management medication care.
